# Providing physical relief for nurses by collaborative robotics

**DOI:** 10.1038/s41598-022-12632-4

**Published:** 2022-05-23

**Authors:** Anna Brinkmann, Conrad Fifelski-von Böhlen, Christian Kowalski, Sandra Lau, Ole Meyer, Rebecca Diekmann, Andreas Hein

**Affiliations:** 1 Assistance Systems and Medical Device Technology, Carl Von Ossietzky University of Oldenburg, Ammerländer Heerstraße 140, 26129 Oldenburg, Germany; 2 Geriatric Medicine, Carl Von Ossietzky University of Oldenburg, 26129 Oldenburg, Germany

**Keywords:** Health care, Health occupations, Risk factors, Engineering

## Abstract

Manual patient handling is one of the most significant challenges leading to musculoskeletal burden among healthcare workers. Traditional working techniques could be enhanced by innovations that can be individually adapted to the physical capacity of nurses. We evaluated the use of a robotic system providing physical relief by collaboratively assisting nurses in manual patient handling tasks. By quantifying kinetic and muscle activity data, it was possible to distinguish two kinds of movement patterns. Highly asymmetric postures and movements corresponded to distinct extremes in lower limb and spine muscle activity data. The use of collaborative robotics significantly reduced maximum force exertion in the caregiving process by up to 51%. Lateral flexion and torsion of the trunk were reduced by up to 54% and 87%, respectively, leading to a significant reduction in mean spine muscle activity of up to 55%. These findings indicate the feasibility of collaborative robot-assisted patient handling and emphasize the need for future individual intervention programs to prevent physical burden in care.

## Introduction

Socio-demographic changes have a high impact on society and healthcare systems worldwide^1^. A decline in birth rates compounds the increase in the life expectancy of the global population. This change is mainly reflected in the growing number of people over 65 years^1,2^. In 2018, for the first time in history, there were more people of this age group than children (< 5 years)^1^. Looking to the future, older people will outnumber adolescents and juveniles (15 to 24 years of age) by 2050^1^, while some regions such as Europe are already finding it difficult to support and care for their elderly population^1,3^. In an international comparison, Germany already has the worst patient-to-caregiver ratio in Europe^4–6^. The imbalance between the provision of health services (e.g., hospital care) and the need for these services is steadily increasing. The number of people over 80 years is expected to triple from 143 million in 2019 to 426 million in 2050^1^, leading to an increased number of people requiring health services and an aging nursing workforce^7^. Consequently, a global shortage of 18 million health workers (50% nurses) is estimated^8^. This will have a significant impact on society and healthcare systems due to global socio-demographic changes and nursing^9^. The latter is a complex issue in every sector of the nursing workforce. Nursing turnover is related to organizational and intrapersonal factors such as workload and mental and physical health^9–17^; there is increasing evidence of these factors' adverse effects on healthcare systems, patients, and nurses^9^. The coronavirus disease, which emerged in 2019 (COVID-19), has affected the entire structure of the world’s population and its healthcare systems, placing an additional tremendous mental and physical burden on healthcare workers^18,19^. Global socio-demographic changes and COVID-19 increase the demand for nurses, rehabilitation programs, and assistive technologies to reduce the enormous mental and physical burden on healthcare workers.

Musculoskeletal disorders are a crucial factor for significant global deterioration in mental health and physical performance^13–17^; they are also closely associated with a high rate of absenteeism and a premature exit from the workforce^13,15^. In this case, low back pain is the main contributor and the most common occupational health problem affecting nurses^10–15,17^. Manual patient handling leads to high mechanical loads in the lower back, and therefore to the development of back complaints, musculoskeletal disorders, and degenerative diseases^13–17^. Asymmetrical lifting, holding, and moving large parts of a patient’s weight strain the caregiver’s lumbar spine through compression, flexion, and rotation; this affects the intervertebral discs by compressive and shear forces^10,12,16,17,20–22^. Indicators of asymmetric loading are lateral flexion and torsional moments of force at the lumbosacral discs resulting from bending or twisting the trunk to the side, lateral arm positions, unequal application of force by both arms, or laterally directed components of the action forces^21^. Musculoskeletal loads can be significantly reduced by minimizing these torque components through ergonomically correct working methods and using technical devices and assistance systems^10,11,17,20–24^. Therefore, to prevent back complaints, it is essential to train nurses to work ergonomically and use technical devices to avoid harmful asymmetric postures and actions such as deep bending or twisting the waist during manual patient transfers and repositioning tasks^16,17,20,21^. The functional ability of nursing staff is another key topic to investigate the multifactorial causality of work-related musculoskeletal disorders and associated lumbar symptoms^21,25–28^. Professional associations recommend that nursing staff do squat training to strengthen the lower limb and back muscles, enabling them to improve their power and functional ability to compensate lumbosacral loads^20,21,26–28^. Hence, the analysis of ergonomic caregiving requires both biomechanical and functional quantification, taking into account the use of assistive devices^29^.

Substantial research has been conducted into detecting the mental and physical factors of occupational health in nursing and developing strategies to prevent job-related back pain^5,10–17,20–24,26–28^. However, recent studies neglect to consider a biomechanical evaluation that takes into account the synthesis of a caregiver’s functional ability and ergonomic working and the use of assistance systems. In addition, there are limitations to scientific contributions that focus on the physical relief of nurses by robotic systems and the power of such systems regarding telepresence and telemanipulation in care^30–33^. For this reason, we developed a “Healthcare Prevention System”^34,35^ that measures the kinematics, kinetics, and muscle activities during manual patient handling^29,30,34–36^. We report the feasibility of a lightweight telepresence and telemanipulation robotic system to provide physical relief by collaboratively assisting nurses while repositioning a patient from a supine to a lateral position in a care bed. This study presents a quantification of lower limb and spine muscle activity in manual patient handling. The proposed technique is the first experimental investigation that evaluates the functional ability of nurses in relation to their physical relief in a supervised ergonomic exercise program using a telepresence and telemanipulation robotic system. We assumed that ergonomic working techniques and the use of a robotic system in manual patient handling tasks lead to a reduction of (a) resulting action forces, (b) lateral flexion and torsional moments of force, and (c) spine muscle activity.

## Methods

### Collaborative robotic system

**Figure 1:**
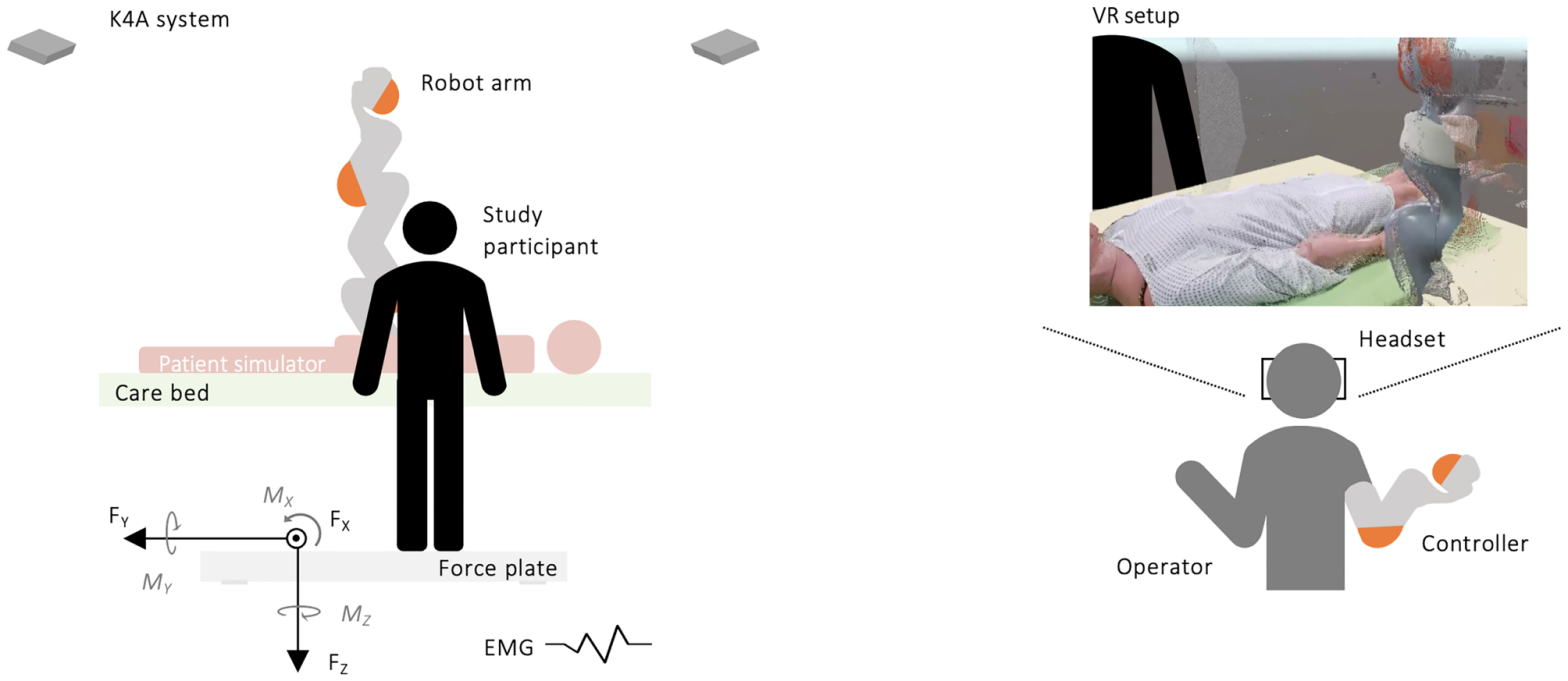
Schematic representation of the study setup of the collaborative robotic system from the side view, including an illustration of the operator’s VR interface (right) to control the robot arm attached to the care bed.

**Figure 2:**
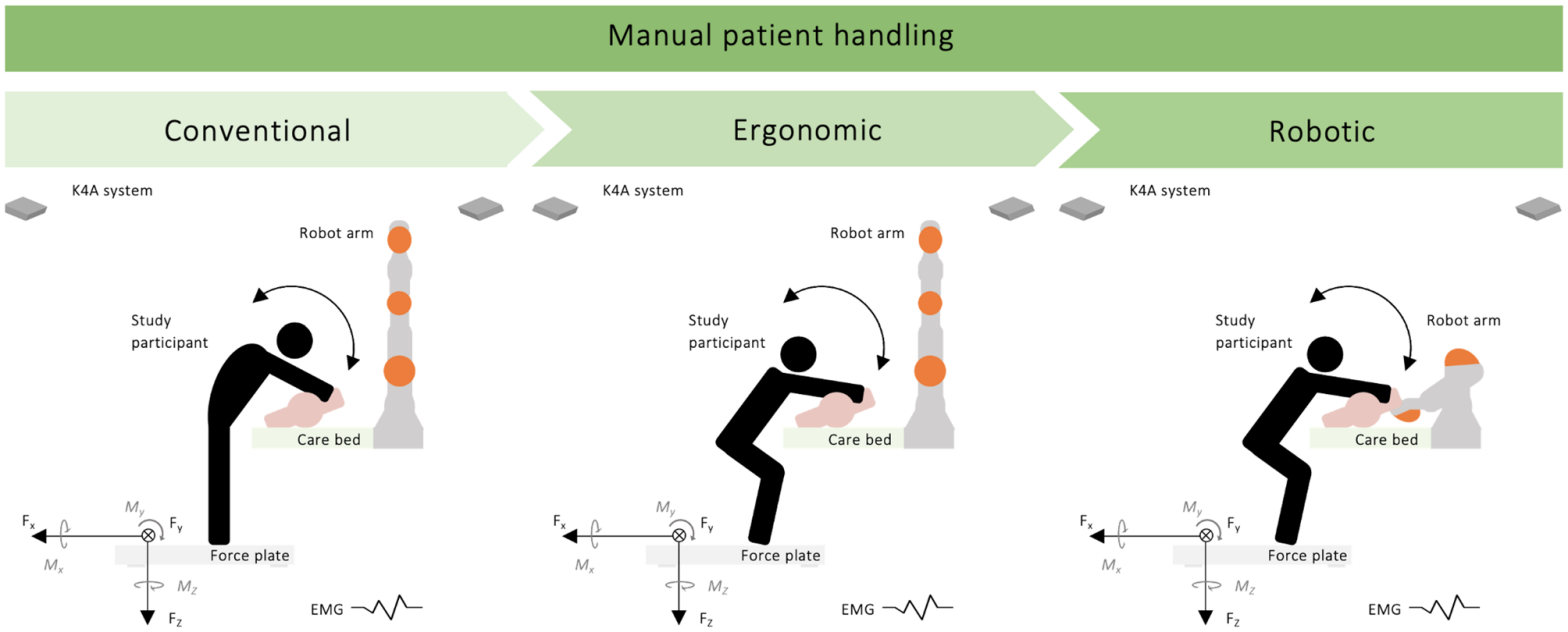
Schematic representation of the experimental design and the manual patient handling tasks, showing the direction of ground reaction forces (F_X_, F_Y_, F_Z_) and respective force plate moments (M_X_, M_Y_, M_Z_). In addition, the study participants were equipped with EMG sensors (Fig. 3).

The robotic system used in this study was a teleoperated robotic solution to help cope with the increasing demand for high-load caregiving. From a distant location, an operator controlled the highly sensitive, anthropomorphic robot arm (KUKA LBR iiwa 7 R800) mounted on the right side of the care bed (Figs. 1 and 2) via a virtual reality (VR) interface (see Supplementary Material, Fig. 1). The robot’s surrounding was observed by a multi-depth camera system^31,35^ that streamed the three-dimensional visual data directly to the operator’s VR headset (Fig. 1). This allowed the operator to participate in care acts such as patient transfer and repositioning tasks, providing collaborative support to the participants using advanced robot equipment.

Although the robotic system has already been described in detail^31^, and this section focuses on its application and potential for physical relief in care, the scope of the system is outlined below.

The operator wore a VR headset (HTC Vive VR System), which enabled him/her to become immersed in the virtual environment within the Unity^®^ engine by Unity Technologies containing the visual data of the multi-depth camera system. With this system the operator was able to control the robotic system for the collaborated manual patient handling task intuitively in real-time^30,31,35^ (Fig. 1). For this purpose, the operator was also equipped with a controller and an elbow tracker^30,31^ (Fig. 1). The controller was used to set the position of the robot’s end effector and the elbow tracker corresponded to the robot’s elbow joint to mimic the operator’s natural arm movement. For the actual robot movement, the operator sent the desired robot positions from within the VR environment to another computer using the Robot Operating System (ROS) Framework. The communication between the Unity^®^ computer and the ROS computer was handled with the ROS#^37^ libraries utilizing the RosBridgeClient. From there, the position signals were forwarded to the robot’s controller via the Fast Robot Interface (FRI). The robot’s positioning was solved from within the Unity^®^ scene with a fast memetic evolutionary inverse kinematics algorithm called BioIK^38^. One advantage of the developed system is that there is no dependence on a specific robot. As soon as an interface for the position specification of the robot at the joint level is available, this robot is suitable for the presented VR control. Necessary is the initial setting of the robot representation in Unity^®^, so that the inverse kinematics of BioIK can be used correctly. Further on, the VR scene showed the calibrated environment of the real world through the 3D camera setup with a low latency of 130 ms and up to 30 fps refresh rate. The coordinate system of the viewpoint was adjusted according to the participant's head position and rotation^31^. Thus, the operator saw the care bed and the quasi-opposite participant (Fig. 1) and used the controller to collaboratively assist during the conducted manual patient handling tasks, thereby moving the patient simulator together (see Supplementary Material, Fig. 1). For this purpose, the multi-depth camera system^31,35^ generated accurate three-dimensional, colored point clouds of the scene. The Microsoft Kinect 4 Azure sensors (K4A) captured depth images and color information. Here, the Amplitude Modulated Continuous Wave (AMCW) Time-of-Flight (ToF) principle was used to record the time for the light exiting the sensor, reflected, and returned to the sensor^31^. Colored three-dimensional point clouds were generated by combining the depth and color information from the K4A’s RGB camera alongside camera-specific calibration parameters using the Near Field of View (NFOV) operation mode at a rate of 30 Hz with a resolution of 640 × 576^31^. Unity 3D was used to visualize the point cloud data^31^. Overall, four K4A were used to eliminate occlusion of important areas caused by people or obstacles at the scene (e.g., nurse, patient, robot). The K4As were installed around the care bed, with the depth images focusing on the main point of interest at the scene. All four K4A were mounted on 1.8-m high tripods covering an area of approximately 2 m × 2 m.

### Experimental design

The investigation was conducted in the fully integrated sensor- and actor-based AAL/Care Laboratory of the Carl von Ossietzky University of Oldenburg to enable a comprehensive measurement-based methodology^34^. A set of successive measurements was taken to assess the physical relief in manual patient handling (Fig. 2). Three different manual patient handling techniques were performed to analyze their potential for load reduction when repositioning a patient simulator from a supine to a lateral position in a care bed, including (1) conventional transfer (non-ergonomic), (2) ergonomic transfer, and (3) transfer in collaboration with a robotic system (Figs. 1 and 2).

**Figure 3:**
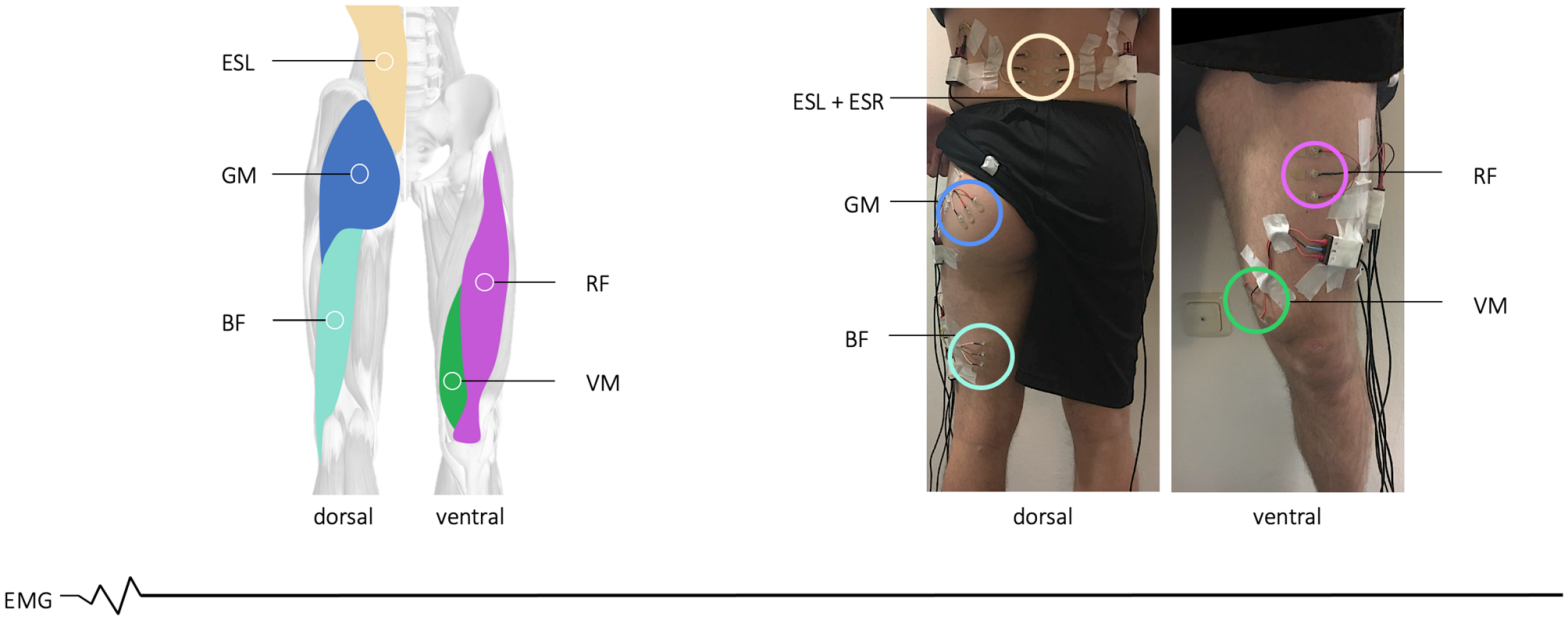
Schematic representation of the EMG sensors (left) attached to the participant’s dorsal and ventral lower limb and to the lumbar spine (right), including the muscle designations. Vastus medialis (VM), rectus femoris (RF), biceps femoris (BF), gluteus maximus (GM), left erector spinae (ESL), and right erector spinae (ESR).

A force plate was used to measure the external ground reaction forces (GRF) and force plate moments (FPM) that occurred^34^ (Figs. 1 and 2). Non-invasive surface electromyography (EMG) recorded the electrical action potentials associated with muscle contraction of the lower limb and spine, providing information on the activation behavior of selected muscle groups (Fig. 3). In addition, a 3D multi-depth camera system^31,35^ was used to control the robotic system and to enable the visual examination of the tasks conducted. To control for experience bias, the participants were introduced to the environment of our laboratory^34^ and asked to practice the patient handling procedures prior to testing. In addition, the participants were given the time to familiarize themselves with the patient simulator and the robotic system. After the familiarization process, bed height adjustment was performed individually, and both the multi-depth camera system and force plate functioning were checked and calibrated. Each task had to be performed on the force plate with the depth cameras focusing on the scene.

Moving a patient from a supine to a lateral position in a care bed is classified as a task associated with the development of degenerative diseases of the lumbar spine^39,40^. Every movement and/or physical burden affecting the human body is transmitted via the sacroiliac joints (SIJ) to the hip joints, and finally to the legs, and vice versa^41^. Hence, extreme shear forces burden the ligaments of the SIJs. Additional muscle groups are essential to compensate for potential overload effects since the ligamentous structures of SIJs are unable to transfer loads in an isolated and effective way from the lumbar spine to the pelvis^41^. SIJ movements and their stabilization mechanisms are influenced by means of lever arms of the muscles of the lower limb and the back extensors, since effective load transfer while preventing shearing of SIJ ligaments is achieved through compression of the joints by ergonomic manual patient handling^41,42^. For this reason, EMG electrodes were placed on the following muscles to record activation behavior according to the tasks performed: vastus medialis (VM), rectus femoris (RF), biceps femoris (BF), gluteus maximus (GM), left erector spinae (ESL), and right erector spinae (ESR) (Fig. 3). For reference purposes, each participant conducted a 60-s sit-to-stand (STS) test^43,44^ (see Supplementary Material, Fig. 2).

### Manual patient handling

**Figure 4:**
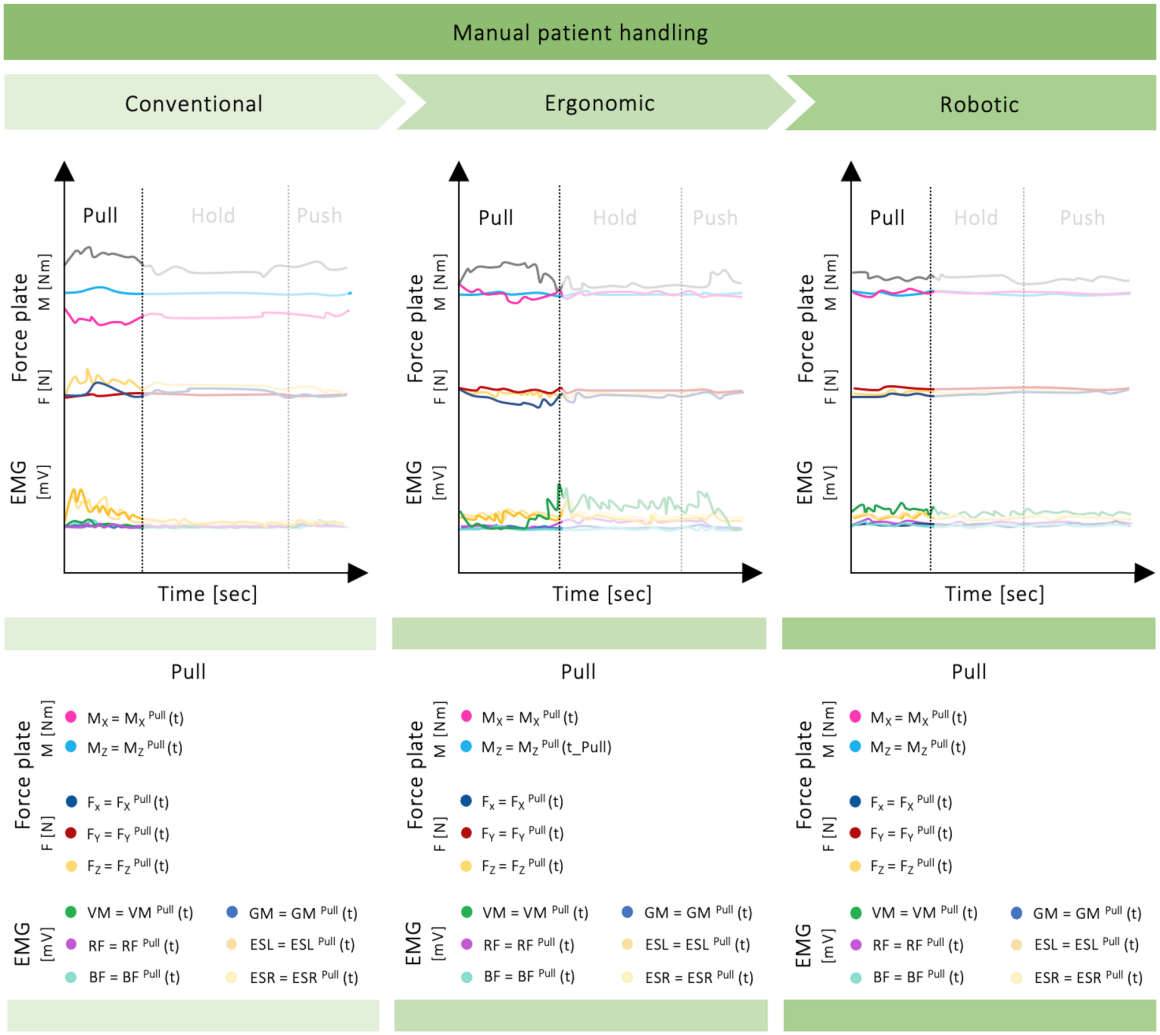
Schematic representation of data collection and processing for the manual patient handling tasks. The ground reaction forces (F_X_, F_Y_, F_Z_), force plate moments (M_X_, M_Z_), and EMG data (VM, RF, BF, GM, ESL, ESR) to be analyzed are visualized for the respective phases of the tasks conducted. Three different manual patient handling techniques were performed (conventional transfer, ergonomic transfer, transfer in collaboration with a robotic system). At the beginning of each task, the patient simulator was already lying in a supine position. Then, the simulator was moved sideward (“Pull”), stabilized for a moment (“Hold”), and returned (“Push”) to the supine position. The “Pull” moving phases were considered for analysis.

Three sets of manual patient handling tasks were conducted (Fig. 2). For our investigation, the Rescue Randy patient simulator (80 kg, 180 cm)^45^ was used instead of a real patient. The participants were instructed to perform all tasks from the left side of the patient simulator and the care bed without stepping off the force plate or supporting themselves on the bed frame. The starting position was standing with both feet on the force plate facing the bed. At the beginning of each task, the patient simulator was already lying in a supine position (Fig. 1). Then, the simulator was moved sideward (“Pull”), stabilized for a moment (“Hold”), and returned (“Push”) to the supine position (Fig. 4). Each transfer task was complete when the participants had returned to an upright position facing the care bed. Overall, three different manual patient handling techniques were performed and described in the following.

#### Conventional

Initially, manual patient handling was performed conventionally (Fig. 2). The participants repositioned the patient simulator without considering ergonomic transfer techniques or biomechanical principles. As a result, the participants were instructed to bend their trunk forward to the patient simulator, grasping under its right shoulder with both hands to powerfully drag the simulator sideward, hold it, and return it to the supine position.

#### Ergonomic

Subsequently, manual patient handling corresponded to an ergonomically and biomechanically optimized repositioning technique (Fig. 2). This technique involved the participants undertaking several pre-positioning activities and preparations to start the caregiving activity correctly. This preliminary step involved limited physical interaction between the participant and the patient simulator. It included pulling up the patient simulator’s knee and bending its leg in preparation for the transfer task. The participants adapted their posture by standing upright parallel to the care bed, with their feet shoulder-width apart. The participants then inclined their trunks to the simulated patient and flexed their knees, shifting their bodies to a stable position. The participants placed their left hand on the simulator’s pulled up knee and their right hand under its right shoulder. They were instructed to ensure ergonomic repositioning of the patient simulator by not using their spine muscles but their leg muscles. Each participant therefore utilized their own body weight and squatted with slightly flexed knees (90°) while moving the patient simulator. The back and neck were stretched, ensuring that the task was not performed with a bent or twisted spine. A hunchback position was to be avoided throughout the task.

#### Robotic

The last step involved the transfer being performed in the same way as in the ergonomic task, but with the support of the robotic system (Figs. 1 and 2). The participant initiated the transfer by slightly lifting the patient simulator so that the robot was touching the shoulder region of the patient simulator, assisting the participant. Once the robot was in contact with the patient simulator, the participant stabilized the simulator and moved it from a supine to a lateral position in the care bed using the remotely controlled robot arm. Consequently, repositioning was conducted collaboratively. That means, the participants and the robot moved the patient simulator together.

### Data collection and processing

#### Participants

The participants were recruited via mailing lists from the University of Oldenburg and a variety of hospitals. The participants were informed that study participation was voluntary, and that they could withdraw from the study at any time. After signing their informed consent, a total of twelve healthy individuals with a nursing background were included in the study. The participants also consented in writing to the publication of identifiable images. A short questionnaire was used to collect demographic data and physical characteristics. Ethical approval was obtained for the study design (ethical vote: Carl von Ossietzky University of Oldenburg, Drs.EK/2019/078). The study was conducted in accordance with the approved guidelines.

#### Measured data

An AMTI Accupower system (AMTI, Watertown, MA, USA), consisting of a force plate and a specialized hardware and software system, was used to measure GRF and FPM. Data was acquired at 200 Hz, displaying GRF in the *X* (mediolateral), *Y* (anteroposterior), and *Z* (longitudinal) direction (F_X_, F_Y_, F_Z_) and respective force plate moments (M_X_, M_Y_, M_Z_) by way of piezoelectric sensors (Figs. 2 and 4). The force plate’s sensitivity was based on 8900 N full-scale F_Z_ capacity and a 12-bit internal analog-to-digital conversion unit^34^.

A 6-channel EMG device from Biovision (Biovision Inputbox and Dasy-Lab 4.010 software) and bipolar surface electrodes (GE Medical/Hellige, 14 mm diameter and 10 mm inter-electrode distance) provided the signals from the muscles of the left lower limb and the lumbar spine under investigation (Figs. 3 and 4). Before applying the electrodes, the skin was gently cleaned with water and then prepared to reduce the skin–electrode impedance. To this end, the hair growing above each muscle was removed with a disposable shaver, abraded with fine sandpaper, and finally cleaned with 70% alcohol^46^. In accordance with SENIAM recommendations^46^, the electrodes were attached to the skin without impeding the range of motion in any position. To validate the EMG signals for crosstalk assessment, low noise values, and proper gain adjustment, various muscle-specific isometric contractions, and muscle palpations were obtained after attachment. The same experienced assessor always placed the electrodes. The EMG signals were sampled at 1000 Hz and amplified (× 2500) using local amplifiers. The signals were then bandpass filtered (10–700 Hz) and sent to the input box using a 14-bit analog-to-digital conversion unit (National Instruments USB-6009).

For further analysis, the data was synchronized by a trigger signal, which involved jumping on the force plate at the start and end of each task.

### Data analysis

The data collected was analyzed using custom functions written in MATLAB (R2021a, MathWorks, Inc., Natick, MA, USA). The first step of data analysis involved visually examining and elaborating the recorded biomechanical data set. After the visual examination, the manual patient handling tasks were synchronized and temporally segmented. Kinematic data of the multi K4A system was only used to visualize the postures and movement strategies adopted by the participants and relate them to the kinetic and muscle activity data set at any time. The viewpoint of the recordings was adjustable, enabling the tasks conducted to be displayed and analyzed from different angles. Note that for the manual patient handling tasks, only the part of the “Pull” moving phases (Fig. 4) were considered for analysis.

Using the force plate software system, it was possible to choose a variety of test sequences. The plain forces were of interest when analyzing indicators for asymmetric loading of the lumbar spine in manual patient handling. For this reason, the GRF measurement system was used with the *Forces Only* test, and the resulting diagrams of GRF and FPM were used for further analysis. F_Z_ force plate data was normalized to each participant’s body mass, and F_X_, F_Y_, F_Z_ were combined to a resultant vector, determining the maximum peak of every manual patient handling task. In this case, we analyzed lateral flexion and torsional FPMs resulting from bending or twisting the trunk to the side, including associated laterally directed GRF components.

Muscle activity was quantified by rectification, root mean square (RMS), and mean value for every muscle and subject. To decrease the inter-individual variability, EMG data was normalized to the peak RMS amplitude recorded during STS (see Supplementary Material, Fig. 3) separately for each muscle and each participant. Moreover, occasional EMG baseline offsets were corrected after visual examination using an offset correction function. EMG data was related to corresponding kinematic and kinetic data in percent. As a result, time axes of collected data were normalized to the period of each task conducted using linear interpolation and divided into respective segments, e.g., “Pull” the patient simulator sideward in manual patient handling tasks (Fig. 4).

### Statistical analysis

First, we evaluated the normal distribution of our data using the Shapiro–Wilk test. Since the sample size was small and the data set included non-normal distributed data, we used the two-sided Wilcoxon signed rank test and the Mann–Whitney test for non-parametric testing to measure significant changes of kinetic and muscle activity data in each of the tasks conducted. We performed inter-group and intra-group comparisons. Effect size (*r*) statistics were provided for both statistical tests. A *p*-value < 0.05 was considered as statistically significant. Statistical analysis was performed using IBM SPSS Statistics software, version 27.0 (IBM Corp., Armonk, NY, USA). All values are reported as means and standard deviations.

## Results

One participant was excluded from data analysis due to missing kinetic force plate data. In total, we analyzed the data sets of 33 manual patient handling tasks from eleven study participants (two male). Individual characteristics of the study population are summarized in Table 1.

**Table 1:**
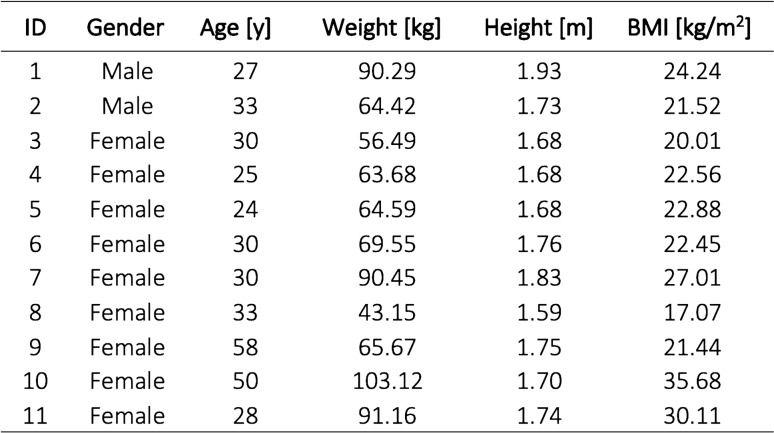
Demographic data and physical characteristics of the study participants.

*BMI* Body Mass Index.

### Force exertion in manual patient handling

In the first step, to differentiate participants’ ability in manual patient handling, we analyzed the kinetic force plate data generated by conventional and ergonomic manual patient handling. Based on literature findings^21^, we assumed that force exertion to pull the patient simulator powerfully sideward during the conventional transfer would be reduced by an ergonomic transfer technique. Each participant's F_X_, F_Y_, and F_Z_ action force components were therefore combined to a resultant vector ($\mid\overrightarrow{r}\mid$). The maximum peak of each participant moving the patient simulator sideward conventionally was then compared to the maximum peak of the ergonomic handling techniques. For further analysis, the participants who were able to reduce resultant action force peaks by acting ergonomically were classified as one group (**A**), and the other participants as another group (**B**) (Table 2).

**Table 2:**
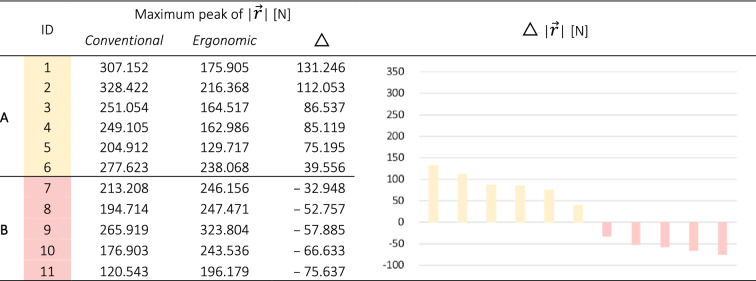
Maximum peak of the resultant ground reaction force vector ($\mid\overrightarrow{r}\mid$) for each study participant and the conventional and ergonomic transfer. The deviation (△) between these tasks is visualized for each participant and group classification (right).

**Figure 5:**
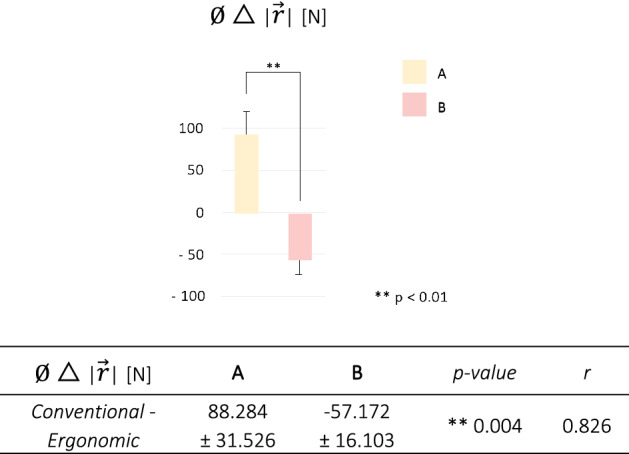
Group classification according to a decrease/increase in the mean $\mid\overrightarrow{r}\mid$ while working ergonomically. The *p*-value and effect size (r) for non-parametric statistical testing using the Mann–Whitney test are also presented.

Highly significant values were found for inter-group comparison in terms of minimizing $\mid\overrightarrow{r}\mid$ when acting ergonomically (*p* = 0.004, r = 0.826) (Fig. 5).

The inter-group comparison did not reach statistical significance, except for the comparison mentioned above, indicating that group **A** and group **B** converge regarding $\mid\overrightarrow{r}\mid$ when using the robotic system (*p* = 0.792, r = 0.110) (see Supplementary Material, Table 1). For this reason, the third transfer mode performed in collaboration with our robotic system to investigate the potential physical relief of all study participants was further analyzed. For this purpose, each participant’s kinetic data for the conventional transfer task was compared to that of the robotic transfer version, using $\mid\overrightarrow{r}\mid$ as described above. In comparison to the conventional repositioning task, all but one study participant reduced the resultant action force peak by using the robotic system (see Supplementary Material, Table 2). Intra-group comparison indicated that $\mid\overrightarrow{r}\mid$ differed significantly concerning the task conducted. Statistical significance of the mean resultant peak value for various manual patient handling tasks was found (Table 3).

**Table 3:**
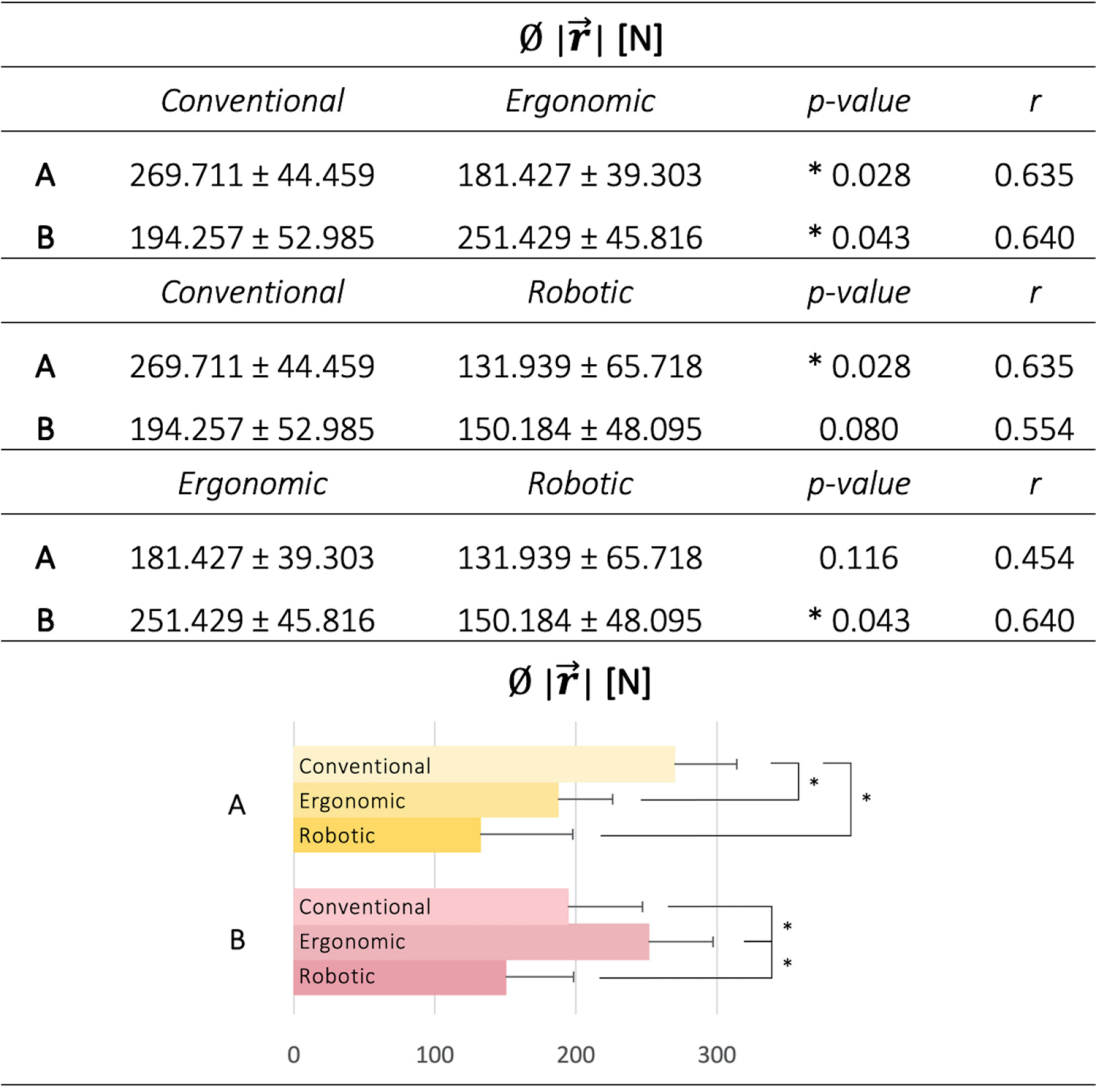
Intra-group comparison of the mean $\mid\overrightarrow{r}\mid$ for all three manual patient handling tasks. The p-values and effect sizes (*r*) for non-parametric statistical testing using the two-sided Wilcoxon signed rank test are presented.

Comparing the conventional task performed by group **B** with the ergonomic version of the patient transfer, significantly higher values were substantiated for the latter (*p* = 0.043, r = 0.640) (Table 3). In contrast, group **A** significantly reduced the mean resultant action force peak by working ergonomically (*p* = 0.028, r = 0.635). Considering the use of the robotic system, the mean maximum peak of $\mid\overrightarrow{r}\mid$ compared to the ergonomic task varied considerably. The use of the robotic system led to a significant reduction for group **B** (*p* = 0.043, r = 0.640) (Table 3). A non-significant reduction was also found for group **A**. When comparing the conventional task with the robotic version, no statistical significance was reached for group **B,** but it was for group **A** (*p* = 0.028, r = 0.635).

Summarizing these results, 5 out of 11 participants could not reduce force exertion in the conventional transfer by using an ergonomic technique. Two groups were therefore identified for further investigation. Group **A** reduced the mean maximum value of the resulting force vector by 32.73% when applying an ergonomic transfer technique and by 51.08% when using the robotic system. In contrast, group **B** could not perform the ergonomic transfer properly, resulting in an increase in $\mid\overrightarrow{r}\mid$ by 29.43%. In this case, the group achieved a significant reduction of 40.27% by using the robotic system.

### Asymmetry in manual patient handling

**Figure 6:**
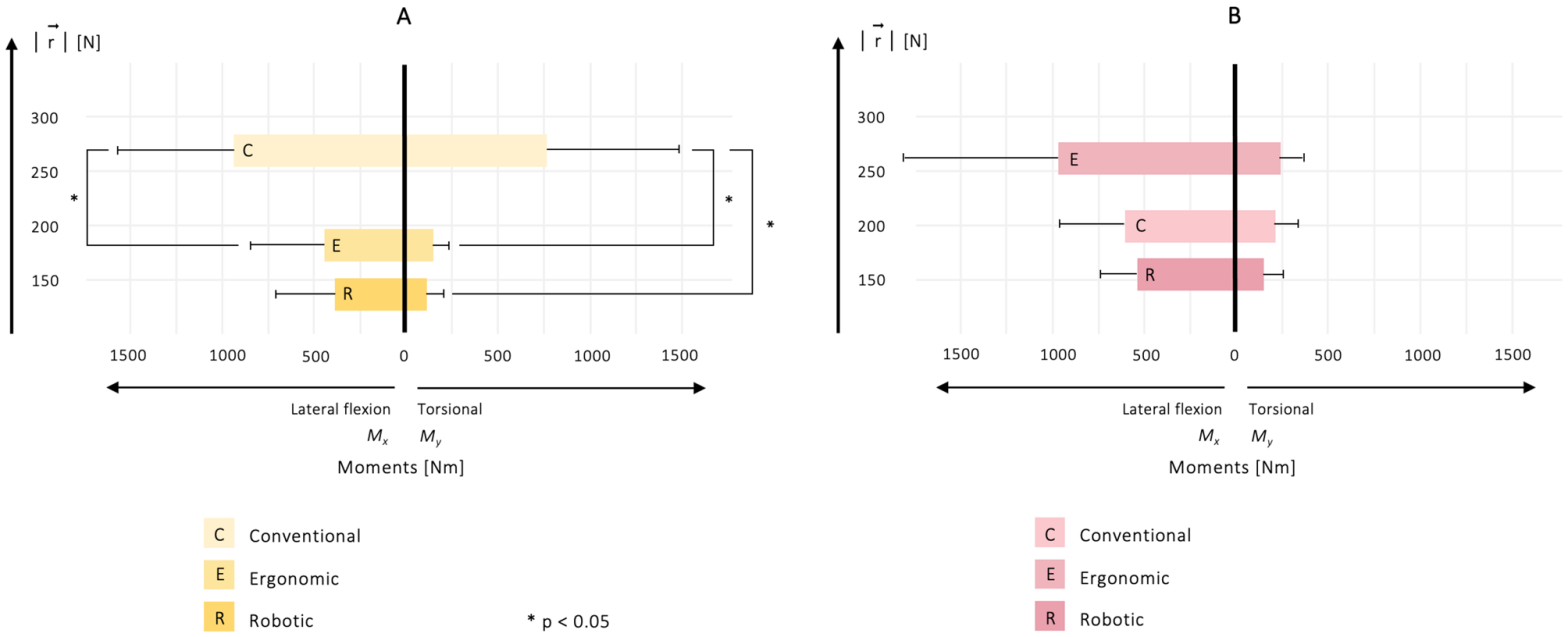
Illustration of the lateral flexion (M_X_) and torsional (M_Z_) moments for both groups and each manual patient handling task in relation to the mean maximum peak value of $\mid\overrightarrow{r}\mid$. The left abscissa shows the moments of lateral flexion; the right abscissa illustrates the moments of torsion. $\mid\overrightarrow{r}\mid$ is depicted by the ordinate. The results are presented with significant comparisons of the two-sided Wilcoxon signed rank test (see Supplementary Material, Table 3).

Force exertion was commonly not symmetrical to the median sagittal plane, resulting from lateral flexion or torsion of the trunk while lifting, holding, and moving large parts of the patient’s weight. To illustrate the level of asymmetry in the tasks conducted, lateral flexion and torsional FPM were analyzed for both groups to further investigate the complexity of biomechanical loading (Fig. 6).

Considering the mean maximum peak values of $\mid\overrightarrow{r}\mid$, the highest value for group **A** was substantiated for the conventional transfer, followed by the ergonomic transfer and the robotic-assisted transfer task (Fig. 6). In contrast, group **B** reached the highest mean maximum peak value in the ergonomic task, followed by the conventional transfer and using the robot arm. For both groups, the collaborative manual patient handling task involving the use of the robotic system led not only to the lowest mean maximum peak values of $\mid\overrightarrow{r}\mid$, but also to the lowest mean maximum lateral flexion and torsional FPM of the trunk. This resulted in fewer asymmetric postures and movements and less force exertion when repositioning the patient simulator to the side using our robotic system. Accordingly, group **A** achieved higher lateral flexion and torsional FPM when performing the conventional task, indicating higher asymmetry in comparison to the ergonomic transfer technique (Fig. 6). For group **B**, higher FPM levels were found for the ergonomic transfer, followed by the conventional task. Statistical significance was reached for group **A** when inducing an ergonomic transfer mode and thereby reducing lateral flexion (*p* = 0.028, r = 0.635) and torsional FPM (*p* = 0.028, r = 0.635), and also when using the robotic system (*p* = 0.028, r = 0.635) (see Fig. 6 and Supplementary Material, Table 3). The inter-group comparison showed no statistical significance (see Supplementary Material, Table 4).

Summarizing these results, lateral flexion and torsion of the trunk increased with an increasing mean maximum peak value of $\mid\overrightarrow{r}\mid$ when moving the patient simulator from a supine to a lateral position in the care bed. In our investigation, the highest degree of asymmetry was found for the conventional task in group **A** and the ergonomic transfer in group **B**. Applying the ergonomic transfer mode, group **A** reduced lateral flexion by 54.14% and torsional FPM by 80.93%. When the robotic system was used, flexion of the trunk was reduced by 51.46% and torsion of the trunk by 86.96%. In comparison, group **B** increased lateral flexion of the trunk by 66.27% with an increasing torsion of 11.09% when working ergonomically. Using the robotic system, FPM resulting from flexion of the trunk was reduced by 42.83% and torsion of the trunk by 35.94%.

### Muscle activity patterns in manual patient handling

**Figure 7:**
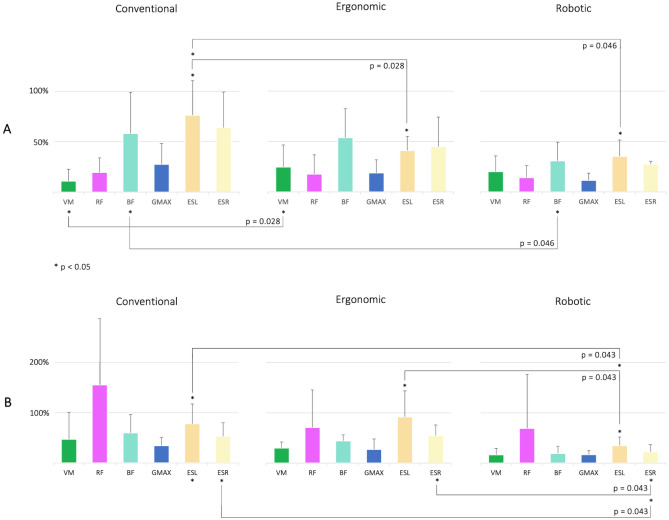
Mean muscle activity data of vastus medialis (VM), rectus femoris (RF), biceps femoris (BF), gluteus maximus (GM), left erector spinae (ESL), and right erector spinae (ESR) normalized by the muscles’ peak RMS amplitude recorded during STS performance (see Methods and Supplementary Material, Fig. 2). The results are presented with significant comparisons of the two-sided Wilcoxon signed rank test for each group and each of the manual patient handling tasks conducted (see Supplementary Material, Table 5).

When moving the patient simulator sideward, a combination of different movement patterns occurred, while maintaining stability through muscle activation. Upon further evaluation, we analyzed the muscle activity data of the lower limb and spine muscles (Fig. 3) for all tasks conducted. Figure 7 illustrates the normalized mean muscle activity data for both groups and each transfer task.

Highly asymmetric postures and movements corresponded to distinct extremes in lower limb and spine muscle activity data for both groups. When the patient simulator was dragged powerfully to the side during the conventional task, concentric work was the main movement type for the spine muscles. For group **A**, this resulted in maximum ESL and ESR muscle activity with maximum support of BF (Fig. 7). In contrast, group **B** exhibited maximum muscle activity for the biarticular thigh muscles RF. When implicating an optimized technique of manual patient handling and using the muscles of the leg instead of the spine for the repositioning task, mean muscle activity data of the spine was reduced significantly in group **A** (ESL: *p* = 0.028, r = 0.635), while VM muscle activity was increased significantly (*p* = 0.028, r = 0.635). In this scenario, group **B** significantly increased spine muscle activity (ESL: *p* = 0.043, r = 0.640). The activity of RF, in particular, was still high, including a higher activity of ESL compared to the conventional transfer. In view of the transfer conducted in collaboration with the robotic system, statistical significance was found for both groups. Group **B** reached a significant reduction of spine muscle activity when comparing both the conventional with the robotic version (ESL: *p* = 0.043, ESR: *p* = 0.043, r = 0.640) and the ergonomic transfer with the robotic transfer task (ESL: *p* = 0.043, ESR: *p* = 0.043, r = 0.640). In contrast, the reduction of spine muscle activity for group **A** was negligible and thus not significant when comparing ergonomic manual patient handling with the robotic-assisted transfer. A significant reduction of BF was achieved when comparing the conventional task with the robotic one (*p* = 0.046, r = 0.575). For both groups, muscle activity patterns of the monoarticular hip muscle GM decreased only moderately in response to the manual patient handling techniques conducted. Inter-group comparison for each task revealed statistical significance for VM and RF when comparing the conventional manual patient handling task (*p* = 0.030, r = 0.661) (see Supplementary Material, Table 6).

Summarizing these results, when using an ergonomic working technique, the mean spine muscle activity of group **A** was significantly reduced by 46.05% (ESL), while thigh muscle activity yielded a significant increase of 127.27% (VM). Moving the patient simulator collaboratively using the robotic system led to a significant reduction of ESL mean muscle activity and BF mean muscle activity by 53.95% and 46.55%, respectively, compared to the conventional transfer task. The biarticular thigh muscle BF was found to have a predominantly higher muscle activity in all three tasks conducted by group **A**. Analyzing the mean muscle activity data of group **B**, a predominantly high biarticular RF muscle activation pattern was yielded in all three transfer modes. The use of the robotic system resulted in a significant reduction in mean muscle activity of the spine muscles. In this case, ESL and ESR mean muscle activity were reduced by 54.55% and 55.77%, respectively, compared to the conventional transfer task.

## Discussion

The participants’ kinetic and muscle activity data was evaluated in relation to the potential physical relief by using three different manual patient handling techniques for moving the patient simulator. Our results confirmed our assumption that (a) resultant action forces, (b) lateral flexion and torsional moments of force, and (c) spine muscle activity can be reduced by properly implemented ergonomic working techniques. In this case, lower limb muscle activity was increased while using leg power instead of moving the patient simulator using the muscles of the spine. For those who were unable to work ergonomically by using the leg muscles, physical relief was achieved by using the robotic system for collaborative assisting.

Previous research has identified that the postures adopted and the action forces and moments exerted were considered the most relevant factors influencing the load on the lumbar spine during manual patient handling^10,12,16,17,20–22^. In addition, professional associations recommend strengthening the lower limb and spine muscles to compensate for high physical stress when moving patients manually. Efforts to provide physical relief for nurses are often based on traditional working techniques, rather than on the use of innovative inventions or the analysis of the physical function of nurses^11,12,16,17,21–24,26–28^. There is a lack of experimental evidence highlighting the specific contribution of the lower limb and spine muscles to manual patient handling techniques. In addition, there is a limitation to studies that investigate the use of robotic systems to protect nurses from musculoskeletal burden. Mukai et al. developed the RIBA robot^32^, a mobile robot equipped with two padded, tactile arms. Thus, its center of mass can be shifted by extending the front tires to independently perform the laborious transfer or lifting tasks for patients weighing up to 63 kg. Hu et al. developed the RoNA robot^33^, which navigates using 3D sensing and perception to lift patients weighing up to 136 kg. Despite their relief potential, both robots are only specialized for one task and thus have the major disadvantage of not promoting mobilization. The patient does not have to make any effort during the transfer. But it is precisely this step that is important during the transfer so that the caregiver experiences the patient's movement limits and encourages him to participate in the movement at his own discretion, which has a positive effect on the body. We used our “Healthcare Prevention System”^34,35^ to collect a biomechanical data set of manual patient handling tasks. For the first time, a lightweight telepresence and telemanipulation robotic system was used to provide physical relief by collaboratively assisting nurses to reposition a patient simulator from a supine to a lateral position in a care bed.

The scenario analyzed in our study was chosen due to its classification by the German Social Accident Insurance Institution for the Health and Welfare Services (BGW)^39^ as an endangering task according to the technical report DIN CEN ISO/TR 12296:2012 (Ergonomics—Manual handling of people in the healthcare sector)^40^. Collaboration with outpatient care services and nurses with extensive professional experience as ergonomic and Kinaesthetics^47^ trainers also revealed that repositioning a patient from a supine to a lateral position in a care bed is a frequent and firmly established activity in everyday nursing care to reach a patient’s back for wound care or cleaning. In addition, this task is a prerequisite step for transferring a patient from a lying to a sitting position at the edge of a bed, e.g., for early mobilization of patients in intensive care units^48^. According to this and previous examinations^21,34,36^, the activity was selected because it was already possible to show biomechanical advantages when comparing a conventional with an ergonomic type of execution in manual patient handling. Hence, this work was designed to ensure a realistic approach without overloading the study participants. Due to ethical and technical reasons—especially regarding using a robotic system—no real patient was recruited for our study. Instead, the patient simulator Rescue Randy^45^ was used in our investigation. Although the patient simulator did not represent a real patient’s body mechanics, a realistic weight (80 kg) and height (180 cm) were considered, as well as the aspect of movable joints, e.g., knees and hip. Thus, our study investigated the repositioning of a non-cooperative patient such as an intensive care patient. Our results should therefore be interpreted within this context.

Since the ligamentous structures of the sacroiliac joints cannot transfer loads in an isolated and effective way from the lumbar spine to the pelvis, additional muscle groups are essential to compensate for potential overload effects in manual patient handling tasks^29,41^. Hence, using the leg muscles is vital in both general physical function and the ability to compensate for lumbosacral loads in care. Thus, our participants conducted different repositioning techniques to examine potential risk factors and to quantify physical relief.

The participants were classified based on individual data evaluation in manual patient handling. One group of study participants (Group **A)** reduced the mean maximum value of the resulting force vector $\mid\overrightarrow{r}\mid$ in the ergonomic and the robot-assisted task. In this case, lateral flexion and torsion of the trunk decreased with decreasing $\mid\overrightarrow{r}\mid$, resulting in a significant reduction of spine muscle activity (ESL) with a significant increase in lower limb muscles (VM, BF) when moving the patient simulator from a supine to a lateral position. In contrast, the other group (group **B**) increased $\mid\overrightarrow{r}\mid$ in the ergonomic transfer task, resulting in an asymmetric movement strategy with higher lateral flexion and torsional FPM compared to the conventional transfer. When utilizing the robotic system to move the patient simulator to the side, lateral flexion and torsion of the trunk decreased with decreasing $\mid\overrightarrow{r}\mid$, resulting in a significant reduction of spine muscle activity (ESL, ESR). In all three transfer modes conducted by group **B**, a predominantly high biarticular RF muscle activation pattern was yielded. The biarticular RF, which is part of the quadriceps, transports moments from the hip to the knee, thereby almost acting as a tendon and redistributing these moments^49^. It can be assumed that the increased muscular activity of RF in group **B** is probably due to increased hip flexion, as asymmetric behavior disrupts balance and control of the movement. This is also indicated by kinematic and kinetic data representing asymmetric movement strategies with significantly higher action forces, higher lateral flexion and torsion of the trunk, and different muscle activity patterns compared to group **A.** With increased hip flexion, high torsional forces are transmitted to the sacroiliac joints since the RF has a distal connection to the tuberosity tibia and a proximal connection to the pelvis. An increased activity of RF may lead to sacroiliac joint pain and may cause lower back disorders^50^. In group **A**, the biarticular thigh muscle BF was found to have a predominantly higher muscle activity in all three tasks. When the lower limb muscles are used to distribute one’s own weight evenly to move the patient simulator, the biarticular BF delivers simultaneous hip extension and knee flexion torques, resulting in a high BF activity pattern. There is evidence indicating that biarticular thigh muscles (BF, RF) increase in response to the disturbance of upper body balance^51^. This might explain our results that BF muscle activity increased when group **A** participants leaned their upper body from forward (grasping the patient simulator) to backward (moving the patient simulator), stretching the back and the neck to perform the tasks without bending or twisting the spine. In contrast, group **B** participants showed high RF muscle activity, which might be due to restoring upper body and lower limb balance. Similar observations were made in the conventional transfer task performed by group **A**. When the transfer tasks were performed both ergonomically and collaboratively with the robotic system, group **A** participants moved less asymmetrically, resulting in a more consistent mean muscle activity. Although group **B** was unable to improve asymmetry by ergonomically moving the patient simulator, the use of robotic assistance yielded a significant reduction of lateral flexion and torsion of the trunk, as well as a significant decrease in spine muscle activity. However, RF muscle activity was still high in this case.

It should be noted that crosstalk from other muscle groups might have influenced the resulting EMG signals. For instance, RF could have been contaminated by vastus intermedius muscle activity^52^. By preparing the skin and placing appropriate surface electrodes in accordance with SENIAM guidelines^46^, the aim was to reduce the influence of crosstalk to a minimum. Although the participants were instructed not to support themselves on the bed frame while performing the tasks, this fact cannot be eliminated from all of the tasks conducted, which may also have led to increased RF activity.

Overall, physical relief in manual patient handling was achieved by minimizing asymmetrical movements and postures through ergonomically correct working methods (group **A**) as well as by using the robotic system (groups **A** and **B**). Looking at the physical characteristics of each group, group **A** participants had a mean age of 28.17 ± 3.43 years, a mean body mass of 68.17 ± 11.62 kg, and a mean body height of 1.74 ± 0.10 m. Group **B** participants had a mean age of 39.80 ± 13.39 years, a mean body mass of 78.71 ± 24.10 kg, and a mean body height of 1.72 ± 0.09 m. The findings in this study highlight the need for individual intervention programs and assistance technologies, which would enhance the underlying manual patient handling techniques to practically systemize the proposed robotic system. One noteworthy issue is that by quantifying lower limb and spine muscle activity in manual patient handling, we contribute to the ongoing research on physical relief of the nursing workforce through functional testing. Even though physical performance changes with age and the prevalence of musculoskeletal disorders increases, younger people are also affected^25^. Low back pain is most common between the ages of 30 and 50 and influences men and women equally^11,20^. According to future forecasts, the number of people with low back pain will increase^15^.

### Limitations

This study was conducted with a small sample size. Therefore, the results and statistical analyses should be interpreted carefully due to the possibility of type II error. To eliminate this error, larger sample sizes are required in future research. In addition, post-hoc effect size analysis—an important index supporting the *p*-value—was not calculated. Some of the normalized mean muscle activation values of group **B** exceeded 100% activation, indicating that the normalization method (STS, see Supplementary Material, Figs. 2 and 3) induced muscle activity less than that of the activities being investigated for manual patient handling. In future research, maximal voluntary contraction could be an additional normalization method. Our study did not control for action forces transmitted by a participant to the bed when touching or moving the patient simulator. Instead, the vector behavior of action forces was analyzed using the ground reaction forces exerted. Force splitting could therefore be another relevant topic in future research.

## Conclusion and implication

In this study, we contribute to the ongoing research on physical relief of the nursing workforce through ergonomic working techniques, including the impact of collaborative robotics. We must adapt our healthcare prevention systems now to identify and minimize risk factors at an early stage, enabling us to offer tailored interventions to prevent physical burden. This study is a contribution to a healthy aging nursing workforce with access to individual intervention programs and assistance technologies in an effort to counteract the already existing shortage of nurses. It is crucial to protect the current nursing workforce and to address growing shortages both now and post-COVID-19.

## Supplementary Information

Supplementary Information.

## Data Availability

The data that support the findings of this study are available from the corresponding author upon reasonable request.
