# Supplementary material for: Providing physical relief for nurses by collaborative robotics
Source: Sci Rep. 2022 May 23;12:8644. doi: 10.1038/s41598-022-12632-4 (PMC9125974; doi:10.1038/s41598-022-12632-4)
Supplement: Supplementary file 1 — Supplementary Information. [file 41598_2022_12632_MOESM1_ESM.pdf]

Providing physical relief for nurses by collaborative robotics

Supplementary Material

Anna Brinkmann,[1,*] Conrad Fifelski-von Böhlen,[1] Christian Kowalski,[1] Sandra Lau,[2] Ole Meyer,[1] Rebecca Diekmann,[1] and Andreas Hein[1]

[1] Assistance Systems and Medical Device Technology, Carl von Ossietzky University of Oldenburg, 26129 Oldenburg, Germany; c.fifelski-von.boehlen@uni-oldenburg.de; christian.kowalski@uni-oldenburg.de; ole.meyer@uni-oldenburg.de; rebecca.diekmann@uni-oldenburg.de; andreas.hein@uni-oldenburg.de

[2] Geriatric Medicine, Carl von Ossietzky University of Oldenburg, 26129 Oldenburg, Germany; sandra.lau@uni-oldenburg.de

[*] Corresponding Author: Carl von Ossietzky University of Oldenburg, Ammerländer Heerstraße 140, 26129 Oldenburg, Germany, anna.brinkmann1@uni-oldenburg.de

Collaborative robotic system

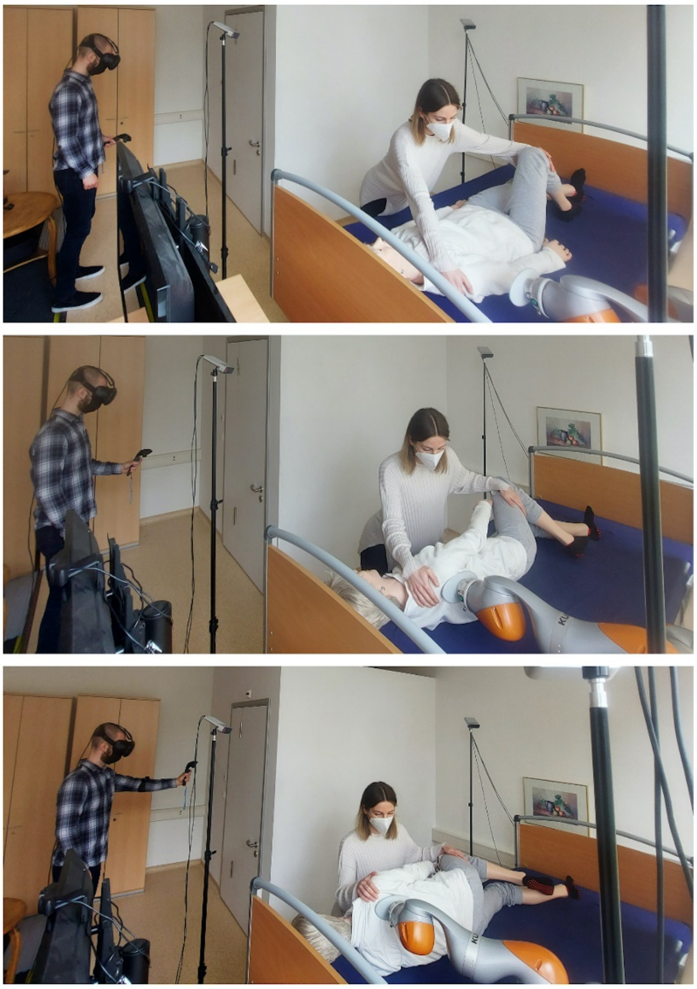

Figure 1. Study setup of the collaborative robotic system, including the operator wearing the VR headset, controller and elbow tracker assisting the participant in moving the patient simulator in the care bed sideward by controlling the robot arm attached to the care bed.

Sit-to-stand testing

The STS covers several components of physical function such as balance or coordination as well as lower limb strength and power [1, 2]. The participants were seated in the center of a standardized armless chair with a seat height of 45 cm (Figure 2). The initial position was a vertical trunk with a stretched back and straight neck. To avoid impulses while rising from or sitting down on the chair, the upper limbs were crossed with bent elbows in the anterior part of the body. The feet were placed next to each other at shoulder width with a knee flexion angle of 90°. All subjects rose to full standing and sat back down with the buttock touching the chair. The participants were instructed to perform the test without stepping off the force plate. After a start command, each participant started to perform as many STS repetitions as possible in 60-sec stopwatch measurements.

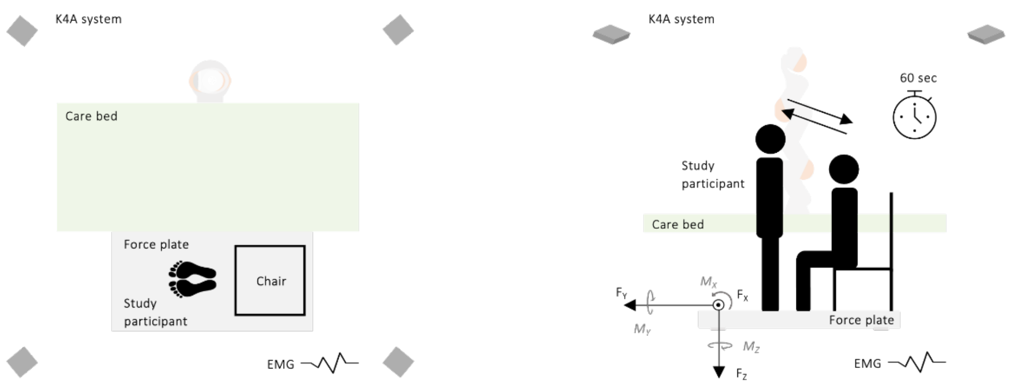

Figure 2. Schematic representation of the study setup for conducting STS testing from the top (left) and side view (right).

Normalization of EMG signals

To decrease the inter-individual variability regarding the manual patient handling tasks conducted, electromyographic (EMG) data were normalized to the peak Root Mean Square (RMS) amplitude recorded during STS for each muscle and each participant separately. The signal of the 60-sec STS test was averaged accordingly, and the RMS peak was then determined by detecting its maximum value.

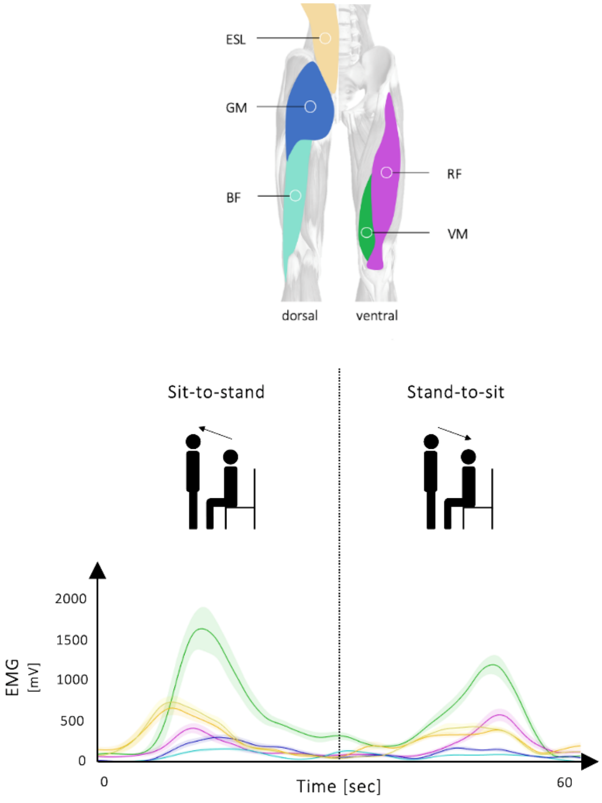

Figure 3. Schematic representation of sit-to-stand and stand-to-sit performances, including RMS mean muscle activity data and the standard deviation of vastus medialis (VM), rectus femoris (RF), biceps femoris (BF), gluteus maximus (GM), left erector spinae (ESL), and right erector spinae (ESR) for one 60-sec STS test and one study participant.

Force exertion in manual patient handling

Table 1 presents inter-group comparison of the maximum peak of resultant ground reaction force vector $|\vec{r}|$ regarding the caregiving tasks conducted. Except for the comparison of the conventional and the ergonomic transfer mode, none reached statistical significance. This indicates that Groups A and B converge regarding $|\vec{r}|$ when using the robotic assistance system ($p = 0.792$, $r = 0.110$).

Table 1. Inter-group comparison of $|\vec{r}|$ for all three manual patient handling tasks. The p-values and effect sizes (*r*) for non-parametric statistical testing using the Mann-Whitney test are presented.

	A	B	*p-value*	*r*
Ø $|\vec{r}|$ [N]				
Conventional	269.711 ± 44.459	194.257 ± 52.985	0.052	0.605
Ergonomic	181.427 ± 39.303	251.429 ± 45.816	* 0.017	0.715
Robotic	131.939 ± 65.718	150.184 ± 48.095	0.792	0.110
⊿ Conventional - ergonomic	88.284 ± 31.526	-57.172 ± 16.103	** 0.004	0.826
⊿ Conventional - robotic	137.772 ± 101.830	44.073 ± 43.998	0.177	0.441
⊿ Ergonomic - robotic	49.488 ± 82.041	101.245 ± 36.550	0.247	0.385

Ø $|\vec{r}|$ [N]

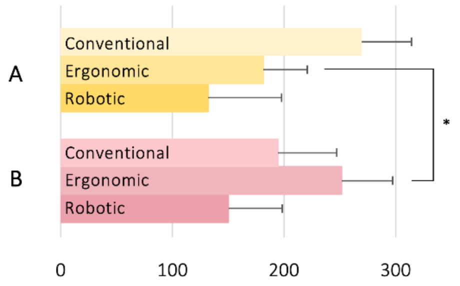

Table 2. Maximum peak of the resultant ground reaction force vector ($|\vec{r}|$) for each study participant and the conventional and robotic assisted transfer. The deviation (Δ) between these tasks is visualized for each participant and group classification (right).

	ID	Maximum peak of $|\vec{r}|$ [N] Conventional	Robotic	Δ
A	1	307.152	62.544	244.608
	2	328.422	58.818	269.604
	3	249.105	100.535	148.570
	4	204.912	178.454	26.458
	5	251.054	204.748	46.306
	6	277.623	186.537	91.086
B	7	213.208	158.826	54.382
	8	194.714	86.824	107.890
	9	265.919	212.820	53.099
	10	176.903	170.809	6.094
	11	120.543	121.642	-1.099

Δ $|\vec{r}|$ [N]

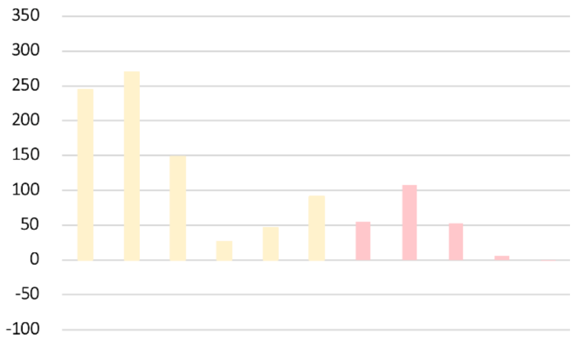

Asymmetry in manual patient handling

Table 3. Intra-group comparison of mean lateral flexion and torsional force plate moments. The p-values and effect sizes (*r*) for non-parametric statistical testing using the two-sided Wilcoxon signed rank test are presented.

	Ø Lateral force plate moments [Nm]			
	Conventional	*Ergonomic*	*p-value*	*r*
A	924.157 ± 631.551	433.067 ± 397.136	* 0.028	0.635
B	582.015 ± 352.135	967.688 ± 828.863	0.500	0.213
	Conventional	*Robotic*	*p-value*	*r*
A	924.157 ± 631.551	448.555 ± 320.508	0.116	0.454
B	582.015 ± 352.135	553.264 ± 197.837	0.893	0.043
	Ergonomic	*Robotic*	*p-value*	*r*
A	433.067 ± 397.136	448.555 ± 320.508	0.917	0.030
B	967.688 ± 828.863	553.264 ± 197.837	0.500	0.213
	**Ø Torsional force plate moments [Nm]**			
	Conventional	*Ergonomic*	*p-value*	*r*
A	768.274 ± 718.435	146.494 ± 94.977	* 0.028	0.635
B	222.570 ± 128.619	247.249 ± 123.577	0.500	0.213
	Conventional	*Robotic*	*p-value*	*r*
A	768.274 ± 718.435	100.221 ± 93.810	* 0.028	0.635
B	222.570 ± 128.619	158.387 ± 109.452	0.345	0.299
	Ergonomic	*Robotic*	*p-value*	*r*
A	146.494 ± 94.977	100.221 ± 93.810	0.600	0.151
B	247.249 ± 123.577	158.387 ± 109.452	0.225	0.384

Table 4. Inter-group comparison of mean lateral flexion and torsional force plate moments. The p-values and effect sizes (*r*) for non-parametric statistical testing using the Mann-Whitney test are presented.

	1	2	*p-value*	*r*
Ø Lateral force plate moments [Nm]				
Conventional	924.157 ± 631.551	582.015 ± 352.135	0.537	0.220
Ergonomic	433.067 ± 397.136	967.688 ± 828.863	0.247	0.385
Robotic	448.555 ± 320.508	553.264 ± 197.837	0.537	0.220
Ø Torsional force plate moments [Nm]				
Conventional	768.274 ± 718.435	222.570 ± 128.619	0.247	0.385
Ergonomic	146.494 ± 94.977	247.249 ± 123.577	0.177	0.441
Robotic	100.221 ± 93.810	158.387 ± 109.452	0.429	0.275

Muscle activity patterns in manual patient handling

Table 5. Intra-group comparison of the normalized mean RMS muscle activity data of vastus medialis (VM), rectus femoris (RF), biceps femoris (BF), gluteus maximus (GM), left erector spinae (ESL), and right erector spinae (ESR) for each task conducted. The p-values and effect sizes (*r*) for non-parametric statistical testing using the two-sided Wilcoxon signed rank test are presented.

		Ø Normalized muscle activity [%]			
		Conventional	*Ergonomic*	*p-value*	*r*
A	VM	11 ± 12	25 ± 22	* 0.028	0.635
	RF	20 ± 14	18 ± 19	0.600	0.151
	BF	58 ± 41	54 ± 29	0.917	0.030
	GM	28 ± 21	19 ± 13	0.249	0.333
	ESL	76 ± 34	41 ± 14	* 0.028	0.635
	ESR	64 ± 35	45 ± 29	0.068	0.527
B	VM	47 ± 53	30 ± 12	0.893	0.043
	RF	127 ± 131	70 ± 75	0.138	0.469
	BF	60 ± 36	45 ± 12	0.715	0.115
	GM	36 ± 16	27 ± 21	0.176	0.428
	ESL	77 ± 39	92 ± 51	* 0.043	0.640
	ESR	52 ± 27	55 ± 21	0.893	0.043
		Conventional	*Robotic*	*p-value*	*r*
A	VM	11 ± 12	20 ± 16	0.207	0.364
	RF	20 ± 14	14 ± 12	0.345	0.272
	BF	58 ± 41	31 ± 18	* 0.046	0.575
	GM	28 ± 21	12 ± 7	0.080	0.506
	ESL	76 ± 34	35 ± 16	* 0.046	0.575
	ESR	64 ± 35	27 ± 3	0.068	0.527
B	VM	47 ± 53	17 ± 16	0.138	0.469
	RF	127 ± 131	69 ± 107	0.080	0.554
	BF	60 ± 36	20 ± 14	0.068	0.577
	GM	36 ± 16	17 ± 8	0.068	0.577
	ESL	77 ± 39	35 ± 17	* 0.043	0.640
	ESR	52 ± 27	23 ± 14	* 0.043	0.640
		Ergonomic	*Robotic*	*p-value*	*r*
A	VM	25 ± 22	20 ± 16	0.345	0.272
	RF	18 ± 19	14 ± 12	0.753	0.092
	BF	54 ± 29	31 ± 18	0.075	0.514
	GM	19 ± 13	12 ± 7	0.080	0.506
	ESL	41 ± 14	35 ± 16	0.463	0.212
	ESR	45 ± 29	27 ± 3	0.068	0.527
B	VM	30 ± 12	17 ± 16	0.225	0.384
	RF	70 ± 75	69 ± 107	0.686	0.128
	BF	45 ± 12	20 ± 14	0.068	0.577
	GM	27 ± 21	17 ± 8	0.144	0.462
	ESL	92 ± 51	35 ± 17	* 0.043	0.640
	ESR	55 ± 21	23 ± 14	* 0.043	0.640

Table 6. Inter-group comparison of the normalized mean RMS muscle activity data of vastus medialis (VM), rectus femoris (RF), biceps femoris (BF), gluteus maximus (GM), left erector spinae (ESL), and right erector spinae (ESR) for each task conducted. The p-values and effect sizes (*r*) for non-parametric statistical testing using the Mann-Whitney test are presented.

		A	B	*p-value*	*r*
Ø Normalized muscle activity [%]					
Conventional	*VM*	11 ± 12	47 ± 53	* 0.030	0.661
	RF	20 ± 14	127 ± 131	* 0.030	0.661
	BF	58 ± 41	60 ± 36	0.914	0.064
	GM	28 ± 21	36 ± 16	0.537	0.220
	ESL	76 ± 34	77 ± 39	0.931	0.055
	ESR	64 ± 35	52 ± 27	0.556	0.222
Ergonomic	*VM*	25 ± 22	30 ± 12	0.662	0.165
	RF	18 ± 19	70 ± 75	0.082	0.551
	BF	54 ± 29	45 ± 12	0.762	0.128
	GM	19 ± 13	27 ± 21	0.662	0.138
	ESL	41 ± 14	92 ± 51	0.052	0.605
	ESR	45 ± 29	55 ± 21	0.730	0.148
Robotic	*VM*	20 ± 16	17 ± 16	0.931	0.055
	RF	14 ± 12	69 ± 107	0.177	0.441
	BF	31 ± 18	20 ± 14	0.257	0.386
	GM	12 ± 7	17 ± 8	0.413	0.295
	ESL	35 ± 16	35 ± 17	0.931	0.055
	ESR	27 ± 3	23 ± 14	0.905	0.074

References

[1] Csuka, M. and McCarty, D.J. Simple method for measurement of lower extremity muscle strength. AM. J. Med. 78, 77-81 (1985).

[2] Hardy, R.; Cooper, R.; Shah, I.; Harridge, S.; Guralnik, J.; Kuh, D. Is chair rise performance a useful measure of leg power? Aging Clin Exp Res 2010, 22(5-6), pp. 412-8. doi: 10.1007/BF03324942
